# Aggressive Posterior Retinopathy of Prematurity: Long-Term Outcomes Following Intravitreal Bevacizumab

**DOI:** 10.3389/fped.2022.778585

**Published:** 2022-02-11

**Authors:** Ameay V. Naravane, Peter J. Belin, Shaina Rubino, Polly A. Quiram

**Affiliations:** ^1^Department of Ophthalmology and Visual Neurosciences, University of Minnesota, Minneapolis, MN, United States; ^2^VitreoRetinal Surgery, Physician Associates, Minneapolis, MN, United States; ^3^North Carolina Retina Associates, Chapel Hill, NC, United States

**Keywords:** aggressive posterior retinopathy of prematurity, APROP, bevicizumab, intravitreal injection, laser, premature (babies), neurodevelopmental outcomes, ophthalmic outcomes

## Abstract

**Purpose:**

The purpose of this study is to review the neonatal and early childhood course of children who were treated with intravitreal bevacizumab for APROP and identify any long term limitations these children face years after treatment.

**Methods:**

This retrospective consecutive case series reviewed both ophthalmologic and pediatric medical records to determine ocular and neurologic function following treatment with a single injection of intravitreal bevacizumab (IVB) for APROP. Patient records were reviewed to identify the gestational age, average birth weight, gender, post-menstrual age (PMA) at the time of injection, regression status, rescue therapy events, final visual acuity, final refraction, ophthalmologic diagnoses and complications, neurologic diagnoses, and duration of follow up.

**Results:**

The study included 43 eyes from 13 male and 9 female children. The average gestational age was 24 weeks and average birth weight was 625.2 grams. The average follow-up was 4.08 years (range: 1.85–7.36 years). The average PMA at time of bevacizumab injection was 35.59 weeks. Thirty-five eyes eventually received laser photocoagulation at an average PMA of 53.17 weeks. All eyes in this study demonstrated regression without progression to retinal detachment. At last follow up, 67% (29/43) of eyes were able to discern letters or shapes, with an average visual acuity of 20/37. 16 (72%) children were diagnosed with perinatal neurological disorders. 59% (*n* = 13) developed chronic neurological impairment, 77% (*n* = 10) of whom developed neurodevelopmental delay. Several infants were diagnosed with endocrine disease or genetic syndromes.

**Conclusions:**

Extreme prematurity is associated with significant morbidity. Nearly all infants (92%) who developed chronic neurologic disease were diagnosed with neurologic disease during the perinatal period. Intravitreal bevacizumab, often with adjuvant photocoagulation, led to regression without detachment in 100% of eyes, with most verbal children retaining functional vision.

## Introduction

Retinopathy of prematurity (ROP) is one of the most common causes of blindness in children. It is a vasoproliferative disorder that occurs in two stages. First, preterm birth exposes infants to a relative hyperoxic environment. Hyperoxia decreases production of growth factors including vascular endothelial growth factor (VEGF) in the retina, leading to delayed vascular maturation. Second, as the retina matures, the increased metabolic activity overwhelms the oxygenation supply from the existing vascular supply resulting in retinal ischemia. This in turn results in increased production of VEGF leading to abnormal neovascular proliferation (1, 2).

Aggressive Posterior Retinopathy of Prematurity (APROP) is a rapidly progressing form of ROP characterized by its posterior location, presence of plus disease, and ill-defined features (3, 4). These eyes tend to have a poorer prognosis with a retinal detachment rate as high as 45% (5).

Laser photocoagulation was initially established as the standard of care by the Early Treatment for ROP (ETROP) Study (6). However, intravitreal bevacizumab (IVB), an anti-vascular endothelial growth factor (anti-VEGF) agent, was later shown to be effective in treating ROP through the Bevacizumab Eliminates the Angiogenic Threat of ROP (BEAT-ROP) trial (7). Today, although both laser and intravitreal bevacizumab (IVB) continue to be used in initial management of type 1 ROP, bevacizumab has become a critical component of the management of APROP (8). Bevacizumab is preferred to laser photocoagulation because it is a less invasive, shorter procedure, and has a decreased recurrence rate of zone 1 ROP (7). Moreover, laser photocoagulation in APROP can present technical challenges due to the presence of a persistent tunica vasculosa lentis, hazy vitreous, and difficulty in distinguishing the border between vascularized and non-vascularized retina (9, 10).

However, robust randomized controlled trials regarding the long term ophthalmic and systemic outcomes of intravitreal bevacizumab are lacking. There is longstanding concern that anti-VEGF medications have adverse systemic effects on these premature infants, particularly their neurodevelopmental outcomes. The purpose of this study is to review the neonatal and early childhood course of children who were treated with intravitreal bevacizumab for APROP and identify any chronic functional limitations these children face years after treatment.

## Methods

This retrospective consecutive case series reviewed both ophthalmologic and pediatric medical records to determine ocular and neurologic function following treatment with intravitreal bevacizumab for APROP. The protocol was exempted for review by the VSRF Salus Institutional Review Board and was compliant with the Health Insurance Portability and Accountability Act. The study adhered to the tenets of the Declaration of Helsinki.

A total of 43 eyes of 22 premature infants born over a 2-year period met inclusion criteria. The study included 13 males and 9 females. All infants were diagnosed with APROP and treated with a single intravitreal injection of bevacizumab. Patient records were reviewed to identify the gestational age, average birth weight, gender, post-menstrual age (PMA) at the time of injection, regression status, rescue therapy events, final visual acuity, final refraction, ophthalmologic diagnoses and complications, neurologic diagnoses, and duration of follow up. All neuro-developmental testing performed on this cohort was assessed by Bayley Scales of Infant and Toddler Development, third edition.

### Treatment

Laser photocoagulation was used as adjunctive therapy for patients in this cohort. The typical treatment practice pattern observed by the senior author of this study include initial treatment with IVB at an average age of 35 weeks. Adjuvant treatment with laser photocoagulation was also used. Infants treated with intravitreal bevacizumab received injections in the neonatal intensive care unit. The eyes were prepped in the typical manner for intravitreal injections. Tetracaine drops were given for anesthesia, ocular adnexa were sterilized with betadine swabs, betadine was placed on the ocular surface, 0.0625 mg of intravitreal bevacizumab was injected 1 mm from the limbus using a short 32 g needle.

**Figure 1:**
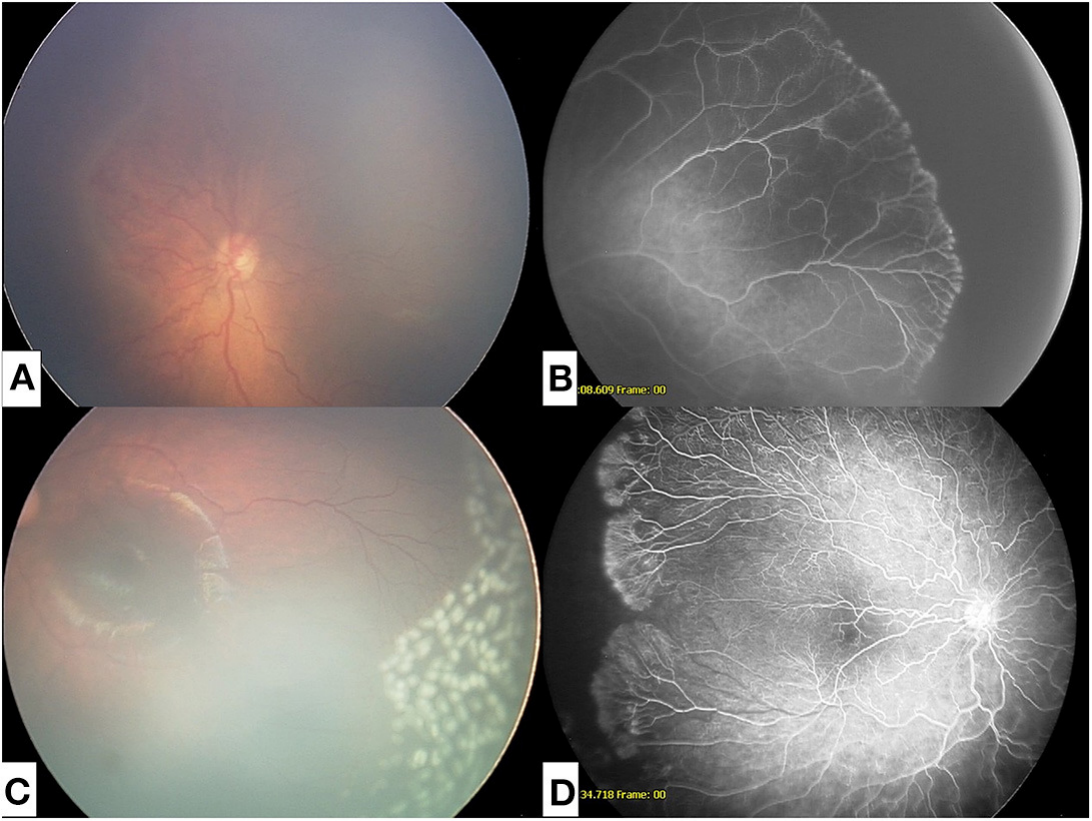
**(A)** Fundus photograph at time of diagnosis of APROP. **(B)** Fluorescein angiogram of APROP demonstrating peripheral non-perfusion. **(C)** Fundus photograph of regressed APROP following intravitreal injection with bevacizumab and modified peripheral photocoagulation. **(D)** Fluorescein angiogram of reactivated APROP following intravitreal bevacizumab.

For patients who received laser, treatment was performed in the operating room under general anesthesia. Laser photocoagulation was performed using indirect ophthalmoscopy in an intentionally less confluent pattern to the peripheral avascular zone, as previously described in Mammo et al. (11) Figure 1 illustrates a sample patient at time of APROP diagnosis and after treatment with both IVB and laser.

## Results

The study included 43 eyes from 13 male and 9 female children. The average gestational age was 24 weeks (range: 23–27 weeks) and average birth weight was 625.2 grams. The average follow-up was 4.08 years (range: 1.85–7.36 years). The average post-menstrual age (PMA) at time of bevacizumab injection was 35.6 weeks (range: 26–41 weeks). 35 (81%) eyes eventually received laser photocoagulation at an average PMA of 53.2 weeks (39.3–60.9 weeks). One eye received a second laser treatment. All eyes ultimately demonstrated regression, without progression to retinal detachment.

**Figure 2:**
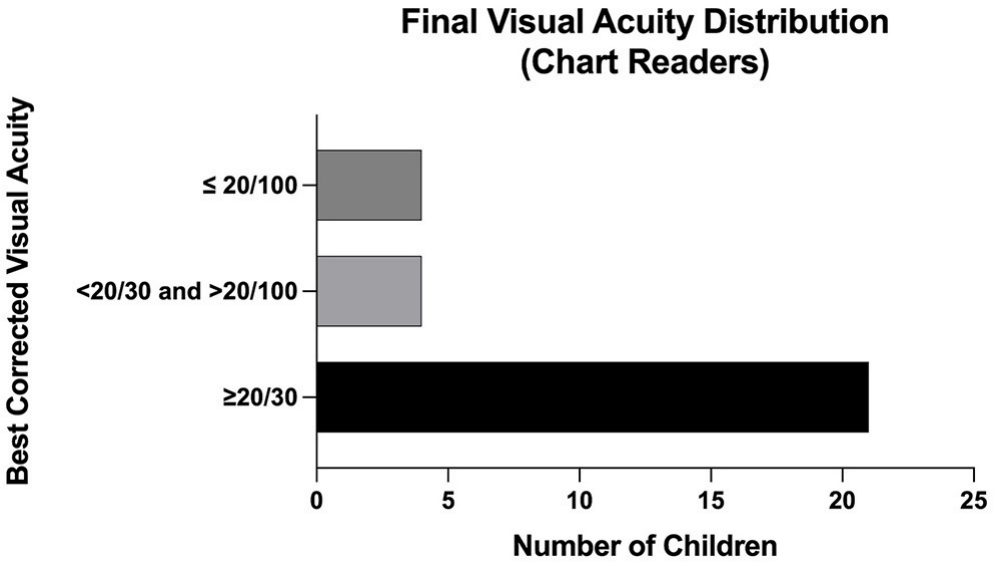
Final Visual Acuity Distribution (Chart Readers) among patients with APROP treated with IVB.

At last follow up, 67% (29/43) of eyes were able to discern letters or shapes, with an average visual acuity of approximately 20/40 (logMAR 0.27). Those unable were largely young or non-verbal. 72% (*n* = 21) of eyes had a visual acuity of 20/30 or better, 14% (*n* = 4) of eyes had a visual acuity between 20/30 and 20/100, and 14% (*n* = 4) of eyes had a visual acuity of 20/100 or worse (Figure 2). Nineteen eyes developed subsequent ocular pathology, which included strabismus (58%, *n* = 11), amblyopia (58%, *n* = 11), nystagmus (21%, *n* = 4), and cataracts (16%, n=3).

Sixteen (72%) infants of the twenty-two infants in this cohort were diagnosed with perinatal neurologic disorders. This was most commonly intraventricular/intracranial hemorrhage (Grade IV) (*n* = 11 infants). One infant developed a thalamic tumor at 2 years old. Perinatal neurological diagnoses acquired by these infants is summarized in Table 1. Thirteen infants (59%) in this cohort ultimately demonstrated chronic neurologic impairment. Of the thirteen, twelve infants (92%) had previously been diagnosed with a neurological disorder in the perinatal period. The most common chronic neurological impairment seen in this cohort was neurodevelopmental delay, seen in 45% (*n* = 10) of infants. Neurodevelopmental delay was tested using Bayley Scales of Infant and Toddler Development, third edition. Chronic neurological impairments sustained by this cohort are summarized in Table 2.

**Table 1:** Perinatal neurological diagnoses in study cohort.

**Perinatal neurologic diagnosis**	**Number of children**
Intraventricular/intracranial hemorrhage (Grade IV)	11
Brachycephaly/plagiocephaly	2
Neonatal cerebral leukomalacia	2
Cleidocranial syndrome	1
Substance withdrawal	1
Velocardiofacial syndrome	1
Other findings	3

**Table 2:** Chronic neurological diagnoses in study cohort.

**Chronic neurologic diagnoses**	**Number of children**
Developmental delay	10
Di-/quadriplegia	1
Cerebral palsy	1
ADHD	3
Autism	3

Several infants were diagnosed with endocrine disease or genetic syndromes including congenital hypothyroidism (*n* = 1), rickets (*n* = 1), type 1 diabetes mellitus (*n* = 1), and Severe Combined Immunodeficiency (SCID) (*n* = 1). 27% (*n* = 6) infants were diagnosed with bronchopulmonary dysplasia/chronic lung disease.

## Discussion

The purpose of this study was to evaluate both long term ophthalmic and neurologic outcomes in infants with APROP who received intravitreal bevacizumab. Our study shows that extreme prematurity is associated with significant neurologic morbidity. Nearly all infants (92%) who developed chronic neurologic disease were diagnosed with neurologic disease during the perinatal period. 73% (*n* = 16) of infants diagnosed with APROP in this study sustained frank perinatal neurologic insult.

The functional and structural outcomes of this case series are promising. Intravitreal bevacizumab, often with adjuvant photocoagulation, led to regression without detachment in 100% of eyes, significantly improved from the 45% retinal detachment rate that has been reported in infants with APROP (5). The combination of IVB with laser treatment is supported by a recent study that found the combination of zone 1 sparing laser photocoagulation and IVB compared to conventional laser photocoagulation alone achieved ROP regression twice as fast (12). With regards to visual acuity, 67% of eyes in our study were able to distinguish letters or shapes and achieved an average acuity of close to 20/40 at an average follow up of 4 years that ranged up to 7 years. As indicated in Figure 2, 72% had a visual acuity better than 20/30 on final follow up. This is comparable to the visual acuity outcomes of infants with APROP treated with laser photocoagulation which reported 81% achieved 20/40 vision or better at an average follow up of 7 years (13).

Management of APROP has shifted toward primary use of intravitreal bevacizumab (8, 14). One reason for this shift is the improved anatomical outcomes with IVB. Similar to the 0% detachment rate in this study, Shah et al. recently reported only a 1% detachment rate in the intravitreal injection cohort compared to 10% in the laser-treated cohort (15). In addition to the reasons alluded to earlier, IVB is a more cost-effective, efficient, and readily available treatment. IVB also allows the retinal vasculature to develop more anteriorly without the permanent peripheral vision loss caused by laser (16). The success rate (defined as complete disease regression) of laser photocoagulation alone in the APROP population have been less promising. The success rate in the literature ranges from 70 to 85% while laser monotherapy in type 1 ROP was above 90% (17). Given the accelerated disease course of APROP, timely treatment is often required. Even when adequate treatment with laser photocoagulation is performed in a timely manner, it often requires 2–3 weeks before the full impact is observed. Treatment with intravitreal bevacizumab, however, has a more rapid onset, typically within 24 h (17). Moreover, 50% of cases treated with adequate laser continue to progress and have poor visual outcomes (2).

Finally, while intravitreal injections are typically performed using only topical anesthesia, laser treatment usually requires general anesthesia. The risks of general anesthesia cannot be understated. In December 2016, the U.S. Food and Drug Administration released a warning that lengthy general anesthesia on children <3 years old may result in neurodevelopmental delay (18, 19). Together, these findings support the primary treatment of APROP with intravitreal bevacizumab.

Our practice pattern often is to follow IVB treatment with adjunctive laser photocoagulation prior to discharge due to our large catchment area and often high risk of poor surgical follow-up. Even the anti-VEGF cohort from Shah et al. mentioned above did require additional laser treatment in 21.4% of infants for disease recurrence. While IVB can begin to provide resolution of APROP within 24 h, adjuvant laser treatment can more permanently treat the anterior avascular retina. In our study, IVB was commonly given at a PMA of 35 weeks, followed by laser at an average PMA 53 of weeks.

Other ophthalmic complications identified in this cohort included cataract formation, nystagmus, amblyopia, and strabismus. The specific incidence of several of these ophthalmic complications among patients with APROP has not been reported in the literature before. For example, 16% of patients in our cohort (*n* = 3) developed cataracts during the follow up period. In comparison, Davitt et al. reported a 1.9% incidence of cataract development by 6 month's corrected age in the ETROP study group (20). 21% of patients in this cohort developed nystagmus. In a large population-based Danish cohort study, the overall incidence of infantile nystagmus in the extremely preterm (<28 weeks GA) was 0.973%, 70% of which were attributable to retinopathy of prematurity (21).

Fifty-eight percentage of our cohort developed amblyopia. The incidence of amblyopia in patients with has previously been reported to be as high as 27.8% in type 1 ROP (22). Finally, 58% of our cohort also developed strabismus. In comparison, a previous study found the rate of strabismus in patients with APROP treated with laser was 40% and those treated with injections was 8% (23).

We suspect that the higher incidence of these ophthalmic complications in our study group compared to what has been previously reported in the literature is due to the longer follow up period in our study. A diagnosis of APROP may also portend more severe ocular comorbidities.

The use of IVB in premature infants remains controversial due to concerns for its possible association with neurodevelopmental delay. Many have also raised concerns regarding the use of IVB because VEGF has an important role in organogenesis of other organs including the lungs and kidneys. This is of particular concern in infants with APROP, a population subset that tends to be younger (gestational age <30 weeks), weigh less (birth weight <1,000 g), and have more medical comorbidities (17, 24). Previous studies have shown that 0.625 mg intravitreal injection of bevacizumab can last in the systemic circulation of premature infants for at least 8 weeks (25). There is some concern that use of IVB after laser treatment could potentiate further systemic spread.

Two major studies have suggested that intravitreal bevacizumab injections are associated with neurodevelopmental delay. Morin et al. showed that infants who received intravitreal bevacizumab were 3 times more likely to develop severe neurodevelopmental disabilities when compared to those who only received laser (26). Natarajan et al. reported higher mortality and worse cognitive outcomes in infants receiving IVB (27). However, in both studies, IVB was primarily given to sicker infants (19, 28, 29). Infants in the IVB group in the Morin et al. had more severe ROP at baseline. Infants in the IVB group in Natarajan et al. were born at a younger gestational age, had a lower median birth weight, required a longer length of ventilator support.

A growing body of evidence has shown no significant difference in neurodevelopmental outcomes between infants treated with IVB and those treated with laser (15, 28–31). A recent meta-analysis of eight studies published by Tsai et al. revealed that patients receiving IVB for ROP were not at an increased risk of severe neurodevelopmental impairment (32).

In the authors' view, the current evidence suggests a low risk of neurodevelopmental delay from IVB in infants. One confounding factor in these studies is that many infants who develop ROP are at risk for neurodevelopmental delay due to other independent risk factors (28, 33). ROP zone itself has been shown to be a risk factor for neurodevelopmental delay (33). On the other hand, the severity of ROP has not been shown to be associated with neurodevelopmental delay (22).

While these data are reassuring, a cautious approach to treatment with IVB is prudent. This is even more important the more vulnerable APROP population. One way to mitigate possible systemic effects of IVB is to reduce bevacizumab dosage to the lowest dose necessary. Clinical trials aimed at assessing the lowest effective dose of bevacizumab with the goal of mitigating impact on organogenesis systemically are ongoing (34).

Given that most infants who had chronic neurological impairment in our study had a prior neurological insult in the neonatal period, we would argue that optimizing these infants' visual potential is not only helpful from an ophthalmic perspective but from a neurologic perspective as well. The role of adequate vision in neurodevelopment cannot be understated.

Finally, our study also revealed several infants who were diagnosed with endocrine or genetic syndromes such as congenital hypothyroidism or SCID. While it is unlikely that these are related to the administration of IVB and more likely related to their extremely premature status, additional studies are required to further elucidate this association.

Limitations of this case series include its retrospective nature and limited sample size. This study is also limited by the lack of a control group to compare long term ophthalmic and neurologic outcomes.

In summary, the results herein indicates that infants with APROP had poor long term neurological outcomes. However, treatment with IVB and adjuvant photocoagulation, resulted in good structural and functional ophthalmic outcomes.

## Meeting Presentation

This work was previously as a poster at ARVO 2019 Annual Meeting.

## Data Availability Statement

The datasets presented in this article are not readily available because data is proprietary to the institutions from which they were collected. Requests to access the datasets should be directed to Ameay Naravane, ameay.naravane@gmail.com.

## Author Contributions

AN: manuscript drafting and revisions. PB and PQ: manuscript review. SR: data collection. All authors contributed to the article and approved the submitted version.

## Funding

This work was supported by the VitreoRetinal Surgery Foundation.

## Conflict of Interest

The authors declare that the research was conducted in the absence of any commercial or financial relationships that could be construed as a potential conflict of interest.

## Publisher's Note

All claims expressed in this article are solely those of the authors and do not necessarily represent those of their affiliated organizations, or those of the publisher, the editors and the reviewers. Any product that may be evaluated in this article, or claim that may be made by its manufacturer, is not guaranteed or endorsed by the publisher.
